# *Yushania tongpeii* (Poaceae, Bambusoideae), a new bamboo species from north-eastern Yunnan, China

**DOI:** 10.3897/phytokeys.130.34466

**Published:** 2019-08-29

**Authors:** Yu-Xiao Zhang, Xia-Ying Ye, En-De Liu, De-Zhu Li

**Affiliations:** 1 Yunnan Academy of Biodiversity, Southwest Forestry University, Kunming, Yunnan 650224, China; 2 Germplasm Bank of Wild Species, Kunming Institute of Botany, Chinese Academy of Sciences, Kunming, Yunnan 650201, China; 3 Key Laboratory for Plant Diversity and Biogeography of East Asia, Kunming Institute of Botany, Chinese Academy of Sciences, Kunming, Yunnan 650201, China

**Keywords:** New species, north-eastern Yunnan, temperate woody bamboos, *Yushania* sect. *Yushania*

## Abstract

*Yushania tongpeii* D.Z.Li, Y.X.Zhang & E.D.Liu, a new species of the temperate bamboo tribe Arundinarieae (Poaceae: Bambusoideae), is described and illustrated from north-eastern Yunnan, China. *Yushania tongpeii* is characterised by taller branching from nodes 1–2 m above the ground, usually three branches at the node, sparse purple spots and thin white powder on the internode, densely purple-spotted culm sheaths, glabrous margins of culm sheaths and tomentose leaf ligules. Based on the morphological features, this new species is assigned to section Yushania.

## Introduction

*Yushania* P.C.Keng, (1957) is one of the largest genera of the tribe Arundinarieae (i.e. the temperate woody bamboos) (Poaceae, Bambusoideae). It consists of more than 80 species, which are mainly distributed in the mid-elevation mountains and subalpine areas (1000–3800 m alt.) of East and Southeast Asia, with the centre of diversity situated in south-western and south-eastern China (Keng 1957, Keng and Wang 1996, Ohrnberger 1999, Li et al. 2006, Vorontsova et al. 2017, Yi et al. 2008, 2017), especially the biodiversity hotspot Mountains of Southwest China. These bamboos are dominant elements for the understorey vegetation of the forest ecosystem. More than 70 species of *Yushania* have been described in China (Yi et al. 2008, 2017), some of which are the staple food of the giant panda (Yi and Jiang 2010).

Species of *Yushania* are characterised by the long-necked rhizomes, diffuse culms, one to many branches at the node, semelauctant and paniculate inflorescence and three stamens (Li et al. 2006). Most taxa of this genus were described without reproductive features due to infrequent flowering. Only 11 species have inflorescence information in *Flora Reipublicae Popularis Sinicae* (Keng and Wang 1996) and *Flora of China* (Li et al. 2006). The genus *Yushania* is divided into two sections, i.e. Y. sect. Brevipaniculatae T.P. Yi and Y. sect. Yushania, based mainly on the culm height and branch number at the node (Yi 1986, 1995). Sect. Brevipaniculatae is distinguished by taller culms, many and subequal branches at each node and terminal panicles or racemes; while species of sect. Yushania are usually shorter and have 1 branch at the mid-culm node or 1 branch at the lower part of the culm and 3–8 branches at the upper part of the culm and terminal panicles.

During a botanical survey to Sanjiangkou, Wumengshan National Nature Reserve, Daguan County, Yunnan, China in 2016, specimens and relevant DNA samples of several bamboo species were collected. One of them has long-necked rhizomes, usually three branches at the node and occurs at elevations around 2300 m. These characters are typical of the genus *Yushania*. After comparison with specimens of *Yushania* deposited at KUN and some literature (e.g. Keng and Wang 1996, Li et al. 2006, Yi et al. 2008, 2017), we concluded that it did not match any described species of *Yushania*. In order to know more about its habitat, distribution range and morphological features, we revisited Sanjiangkou, Wumengshan National Nature Reserve in September 2018 and more specimens were collected. In this paper, we described it as a new species, i.e. *Yushania tongpeii* D.Z.Li,Y.X.Zhang & E.D.Liu.

## Materials and methods

Observation and measurement of morphological characters of the new species were carried out in the field and the herbarium, based on living plants and specimens. Some characters were observed by stereomicroscope (Leica S6E). Morphological features of the related species (*Yushania oblonga* T.P.Yi, *Y. pingshanensis* T.P.Yi and *Y. straminea* T.P.Yi) were obtained from literature (Keng and Wang 1996, Li et al. 2006, Yi et al. 2008) and specimens deposited at KUN.

## Taxonomy

### Yushania tongpeii

Taxon classificationPlantaePoalesPoaceae

D.Z.Li, Y.X.Zhang & E.D.Liu sp. nov.

DC9AA0C8899457BC88B6FD7D91DB1043

urn:lsid:ipni.org:names:60479346-2

Figures 1 , 2

#### Diagnosis.

*Yushania tongpeii* is morphologically similar to *Y. oblonga*, *Y. pingshanensis* and *Y. straminea*, but can be easily distinguished by having taller branching from nodes 1–2 m above the ground, sparse purple spots on the internode, densely purple spotted culm sheaths, glabrous margins of culm sheaths and tomentose leaf ligules.

#### Type.

CHINA. Yunnan: Daguan County, Wumengshan National Nature Reserve, Sanjiangkou, 28°13'16"N, 103°54'1"E, 2260 m alt., 29 September 2018, *Y.X. Zhang* et al. *18180* (holotype: KUN!; isotype: KUN!).

#### Description.

Rhizomes pachymorph, rhizome neck 17–41 cm long, 0.4–0.6 cm in diameter, solid. Culms 2–5 m tall, 0.8–1.5 cm in diameter; internodes terete, 15–38 cm long, initially sparsely purple-spotted, thinly white powdery, densely below nodes, glabrous; culm wall 2–4 mm thick; nodes inconspicuous; sheath scar prominent, with persistent remains of sheath base. Branching from nodes 1–2 m above the ground, branches usually 3, the base appressed to the culm. Culm sheaths tardily deciduous, oblong, leathery, 1/3 to 1/2 as long as internodes, densely purple-spotted, sparsely setose or glabrous abaxially, margins glabrous; auricles narrowly falcate; oral setae radiate; ligules 1–3 mm tall, truncate, margins entire or shallowly serrate; blades erect or recurved, lanceolate. Foliage leaves 3–5 per ultimate branch, sometimes a slender branchlet with 3–5 foliage leaves extending from the apex of the ultimate branch; sheaths initially sparsely setose and white powdery, glabrescent, green or purple, margins glabrous; auricles narrowly falcate, deciduous; oral setae several, radiate, deciduous; ligules truncate or a little arched, 2–3 mm tall, tomentose abaxially; petiole puberulous, initially white powdery; blades 9.5–21 × 1.2–3 cm, shallowly wavy when dry, glabrous, glaucous abaxially, secondary veins 4–6 pairs, transverse veins conspicuous, apex tapering, margins serrate. Inflorescence unknown.

**Figure 1.**
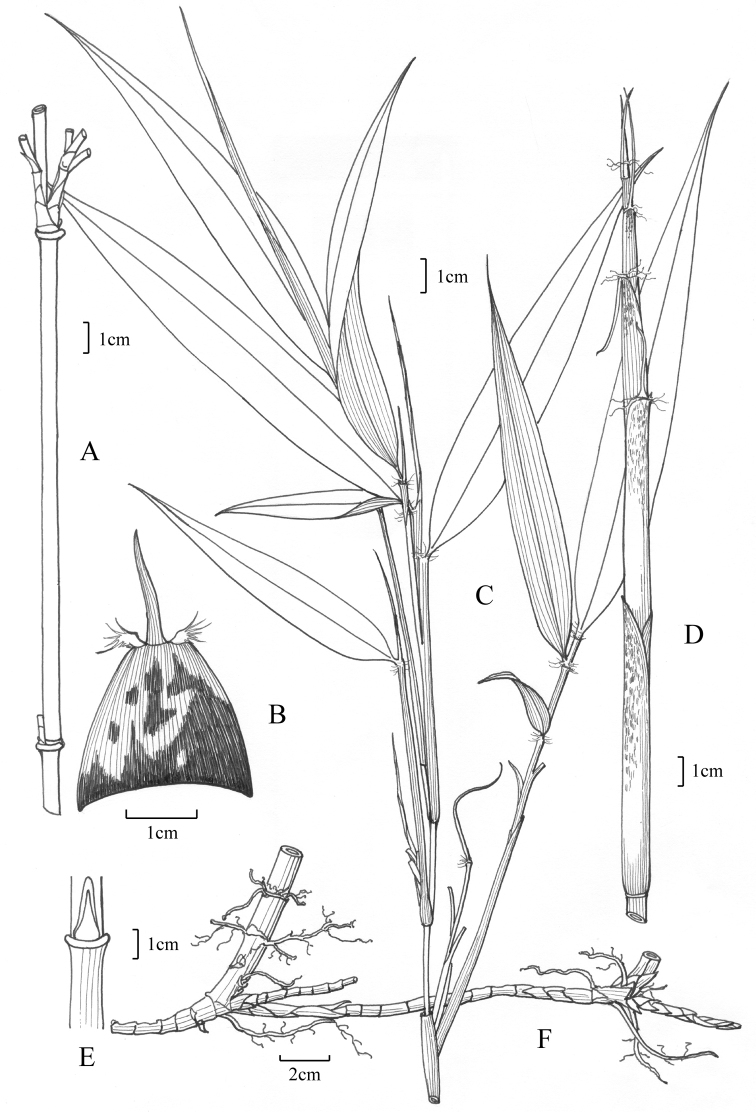
*Yushania tongpeii* D.Z.Li., Y.X.Zhang & E.D.Liu. **A** internode and branches **B** part of the culm sheath, denoting auricles and the blade **C** young leaves **D** new shoot **E** culm bud **F** long-necked rhizome.

**Figure 2.**
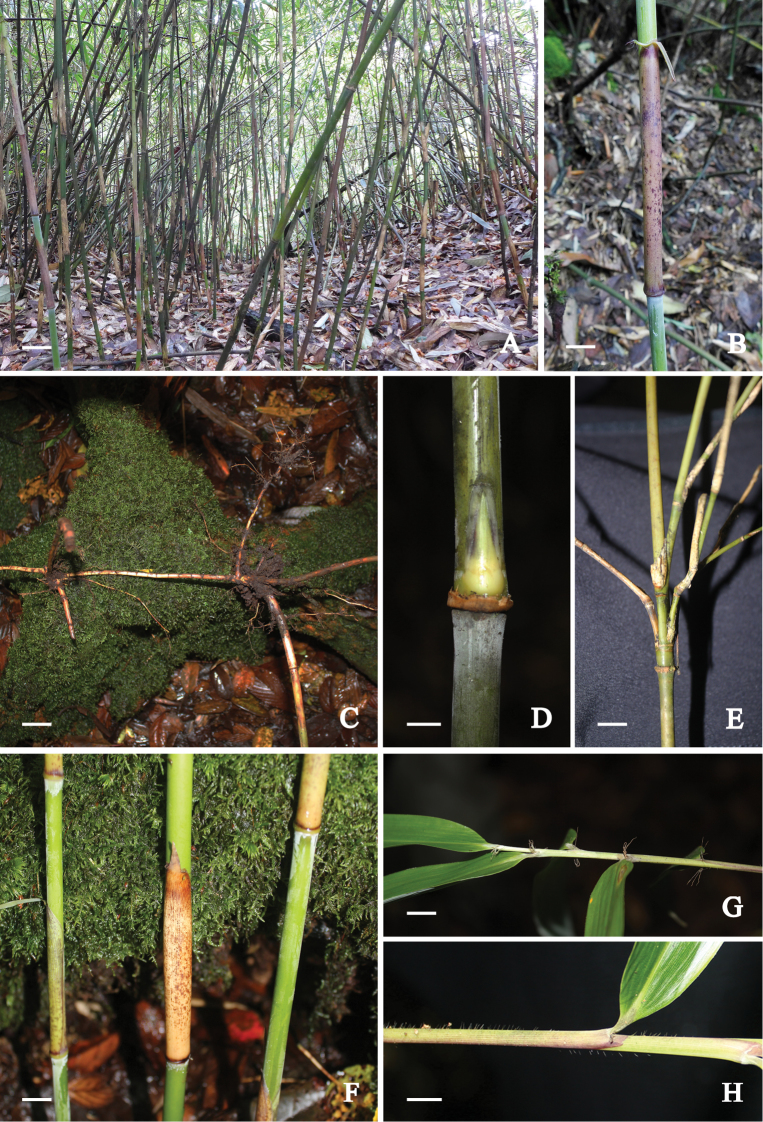
*Yushania tongpeii* D.Z.Li., Y.X.Zhang & E.D.Liu. **A** habitat **B** culm sheath **C** rhizome **D** culm bud **E** branches **F** young culms with culm sheaths **G** foliage leaves **H** leaf sheath. Scale bars: 5 cm (**A**); 2.5 cm (**C**); 2 cm (**B**); 1 cm (**D–G**); 5 mm (**H**).

#### Phenology.

New shoots May to July.

#### Distribution and habitat.

This new species is only found in Daguan County, north-eastern Yunnan, China. It occurs above the upper limit of distribution of *Chimonobambusa tumidissinoda* Hsueh&T.P.Yi ex Ohrnberger in this area, and grows under the evergreen broadleaved forests at an altitude between 2200–2400 m.

#### Etymology.

The specific epithet refers to Professor Tong-Pei Yi (1933–2016), who made great contributions to the taxonomy of the alpine bamboos (particularly in *Fargesia* Franchet and *Yushania* P.C. Keng) in China.

#### Additional specimens examined (paratypes).

CHINA. Yunnan: Daguan County, Wumengshan National Nature Reserve, Sanjiangkou, 28°14'11"N, 103°54'21"E, 2390 m alt., 24 May 2016, *E.D. Liu* et al. *4760* (KUN!), ibid., 28°13'44"N, 103°54'53"E, 2230 m alt., 28 September 2018, *Y.X. Zhang* et al. *18176*, *18177* (KUN!).

## Discussion

The vegetative characters of *Yushania tongpeii*, such as three branches at the upper part of the nodes and medium height culms, are similar to the species of section Yushania, particularly the three species listed in Table 1. All these three species have less than five branches at the node, white powder on the internode or below the node, prominent nodal sheath scar, falcate culm sheath auricles and white powdery petioles (except for *Y. pingshanensis*). However, some subtle features make *Y. tongpeii* distinctive, including sparse purple spots on the internode, densely purple-spotted culm sheaths, glabrous margins of culm sheaths and tomentose leaf ligules. Therefore, this new species should be assigned to section Yushania on the basis of morphology.

**Table 1.** Morphological comparison of *Yushania tongpeii* and related species.

	*Y. tongpeii*	*Y. oblonga*	*Y. pingshanensis*	*Y. straminea*
Culm height	2–5 m	3–4.5 m	1.2–2 m	2–4 m
Culm diameter	0.8–1.5 cm	1–2 cm	0.5–0.75 cm	0.6–1 cm
Internode	15–38 cm long, thinly white powdery, densely below nodes, glabrous	28–40 cm long, initially white powdery, densely below nodes, glabrous	13–35 cm long, a ring of white powder below nodes, glabrous	18–29 cm long, thinly white powdery, densely below nodes, glabrous
Branch complement	Usually 3	1–3 (5)	1–3	1–3
Nodal sheath scar	Prominent	Prominent	Prominent	Prominent, initially retrorsely setose
Culm sheath	Tardily deciduous, densely purple-spotted, sparsely setose or glabrous abaxially, margins glabrous	Persistent, white powdery, glabrous, margins densely yellow setulose	Persistent, densely light yellow verrucose setose abaxially, margins densely ciliate	Persistent, densely setose, margins densely ciliate
Culm sheath auricle	Narrowly falcate	Falcate	Oblong or falcate	Falcate
Culm sheath blade	Erect or recurved, lanceolate	Erect, linear-lanceolate	Recurved, triangular-linear or linear-lanceolate	Erect or recurved, oblong-triangular or elliptic-lanceolate
Leaf number of the ultimate branch	3–5	5–7	5–9	4–9
Leaf sheath	Initially sparsely setose and white powdery, glabrescent, margins glabrous	Glabrous, initially white powdery, margins glabrous	Glabrous, margins glabrous	Glabrous, usually white powdery, margins glabrous
Leaf ligule	Truncate or a little arched, tomentose abaxially	Truncate, glabrous	Truncate, glabrous	arcuate
Petiole	Puberulous, initially white powdery	White powdery	Purple	Puberulous, initially white powdery
Leaf blade	9.5–21 × 1.2–3 cm, glabrous	14–17 × 3.6–4 cm, glabrous	9–17 × 1.3–2.2 cm, glabrous	7–19 × 1.6–2.6 cm, basally grey hairy

Some researchers analysed the diversity and the distribution patterns of endemic seed plants in China (Huang et al. 2014, Huang et al. 2016). The Central Yunnan Plateau Subregion, one of the floristic units in China (Wu et al. 2010), is one of the two centres of Chinese endemic flora (Huang et al. 2016). Wumengshan National Nature Reserve is located at the east edge of the Central Yunnan Plateau Subregion. Therefore, the discovery of *Yushania tongpeii*, which is endemic to Wumengshan National Nature Reserve, gives meaning for studying the diversity of endemic species in this area.

## Supplementary Material

XML Treatment for Yushania tongpeii
